# The Key to Increase Immunogenicity of Next-Generation COVID-19 Vaccines Lies in the Inclusion of the SARS-CoV-2 Nucleocapsid Protein

**DOI:** 10.1155/2024/9313267

**Published:** 2024-05-29

**Authors:** Noe Juvenal Mendoza-Ramírez, Julio García-Cordero, Gaurav Shrivastava, Leticia Cedillo-Barrón

**Affiliations:** ^1^ Departamento de Biomedicina Molecular CINVESTAV IPN, Av. IPN # 2508 Col, San Pedro Zacatenco, Mexico City 07360, Mexico; ^2^ Laboratory of Malaria and Vector Research National Institute of Allergy and Infectious Diseases National Institutes of Health, Rockville, MD, USA

## Abstract

Vaccination is one of the most effective prophylactic public health interventions for the prevention of infectious diseases such as coronavirus disease (COVID-19). Considering the ongoing need for new COVID-19 vaccines, it is crucial to modify our approach and incorporate more conserved regions of severe acute respiratory syndrome coronavirus 2 (SARS-CoV-2) to effectively address emerging viral variants. The nucleocapsid protein is a structural protein of SARS-CoV-2 that is involved in replication and immune responses. Furthermore, this protein offers significant advantages owing to the minimal accumulation of mutations over time and the inclusion of key T-cell epitopes critical for SARS-CoV-2 immunity. A novel strategy that may be suitable for the new generation of vaccines against COVID-19 is to use a combination of antigens, including the spike and nucleocapsid proteins, to elicit robust humoral and potent cellular immune responses, along with long-lasting immunity. The strategic use of multiple antigens aims to enhance vaccine efficacy and broaden protection against viruses, including their variants. The immune response against the nucleocapsid protein from other coronavirus is long-lasting, and it can persist up to 11 years post-infection. Thus, the incorporation of nucleocapsids (N) into vaccine design adds an important dimension to vaccination efforts and holds promise for bolstering the ability to combat COVID-19 effectively. In this review, we summarize the preclinical studies that evaluated the use of the nucleocapsid protein as antigen. This study discusses the use of nucleocapsid alone and its combination with spike protein or other proteins of SARS-CoV-2.

## 1. Introduction

Severe acute respiratory syndrome coronavirus 2 (SARS-CoV-2), a member of the coronavirus family, is an enveloped, positive-sense, single-stranded RNA virus [1]. Its genome encodes four structural proteins: membrane (M), envelope (E), spike (S), and nucleocapsid (N) [2]. This virus is responsible for the coronavirus disease (COVID-19) pandemic and presents with various symptoms, such as cough, fever, and respiratory distress syndrome [3]. From December 2020 to March 2024, the number of confirmed cases of COVID-19 reached 775,251,779 with 7,043,660 reported deaths [4]. According to the coronavirus guidelines, COVID-19 is classified into five types: asymptomatic or presymptomatic, mild, moderate, severe, and critical [5]. The first case tested positive for SARS-CoV-2; however, it did not have symptoms consistent with COVID-19. Patients with mild and moderate patients show fever, cough, headache, muscle pain, and decreased oxygen saturation (SpO2) [5, 6]. Severe shows any of the following symptoms: respiratory distress, respiratory rate ≥30 beats per minute, SpO2 ≤93% at resting, or arterial PaO2/FiO2 ≤300 mmHg. Finally, patients present with any of the following symptoms: respiratory failure, need for mechanical ventilation, shock, and organ failure [6]. Age and comorbidities such as cardiovascular disease, hypertension, diabetes, and cancer increase the risk of death [7]. The rapid development of COVID-19 vaccines and their immediate application is based on insights from studies on SARS-CoV and MERS-CoV, which have contributed to better control of infections and a reduction in deaths [8]. However, since the beginning of the pandemic, being an RNA virus, numerous mutations have emerged in the SARS-CoV-2 genome, particularly in the encoding sequence for spike protein, thereby resulting in different generations of variants [9]. The World Health Organization (WHO) and Centers for Disease Control and Prevention (CDC) have categorized variants into variants being monitored (VBMs), variants of interest (VOIs), variants of concern (VOCs), and variants of high consequence (VOHCs) [10]. Although most vaccines use spike proteins as antigens, a decline in their efficacy against VOCs has been observed [11]. Consequently, second-generation COVID-19 vaccines are now focused on employing different antigens against SARS-CoV-2 to induce a more effective and long-lasting protective immune response that additionally can prevent and combat VOCs. The nucleocapsid (N) protein, is highly conserved among coronaviruses and their variants, and it induces long-term cellular immune responses. Moreover, it has been implicated in the induction of innate memory. Thus, N has emerged as a promising candidate for the development of COVID-19 vaccines [12]. In this review, we summarize the structure, function, and immune responses against the nucleocapsid protein. Significantly, in this review, we intent to include the most of preclinical studies that have explored the use of N protein and its potential to be considered in the elaboration of new vaccines against COVID-19.

## 2. Structure and Functions of SARS-COV-2 Nucleocapsid

**Figure 1:**
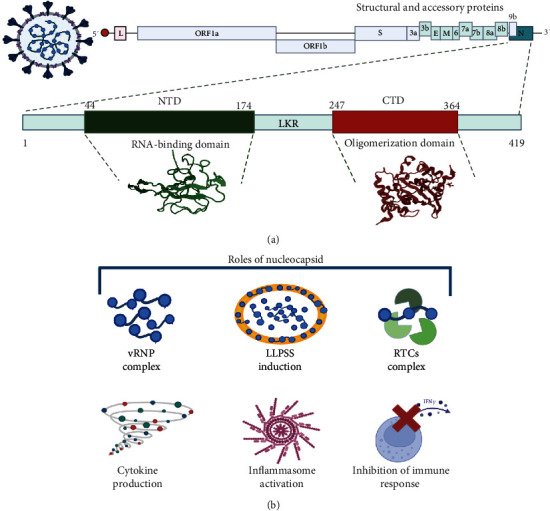
Structure and functions of the nucleocapsid protein. (a) Representation of domains in N. (b) Roles of N protein, formation of vRNP (viral RNA–protein complex), LLPSS (liquid–liquid phase separation), and RTCs (replication transcription complexes), as well as immune responses induced by N.

The N protein is the most abundant protein in the virion and a critical structural component of SARS-CoV-2 with a molecular weight of 46 kDa and RNA-binding capabilities. It is encoded by the ninth open reading frame (ORF) of the SARS-CoV-2 genome, consists of 419 amino acids, and is divided into intrinsically disordered regions (IDRs) and conserved structural regions [13] (Figure 1). The IDRs encompass three modules, the N-arm, the central Ser/Arg-rich flexible linker region (LKR), and the C-tail, whereas the conserved structural regions include two modules: the N-terminal domain (NTD) and the C-terminal domain (CTD). The NTD is essential for RNA binding, whereas the CTD contains a C-terminal dimerization domain [13, 14]. Both the NTD and CTD were flanked by IDRs. NTD are characterized by an abundance of aromatic and basic residues, with tail residues (Asn48, Asn49, Thr50, and Ala51) that are highly flexible and form a binding pocket that effectively interacts with viral RNA. The NTD exhibits a hand-like structure with a protruding basic finger, palm, and wrist [15]. The nucleocapsid is a multifunctional protein involved in viral pathogenesis, replication, and RNA packaging. Additionally, the N protein interacts with the SARS-CoV-2 membrane, promoting aggregation and facilitating the fixation of ribonucleocapsid particles to the viral membrane [16]. N is one of the most immunogenic proteins after the spike protein and is involved in interferon inhibition, RNA interference, apoptosis, and regulation of the viral life cycle [17, 18]. Recently, the N protein was detected in the membranes of infected cells, interacting through its negative charge with glycosaminoglycans such as heparan sulfate and heparin. However, the precise mechanism by which these proteins associate with membranes remains unknown [19]. N proteins undergo several posttranslational modifications including phosphorylation, methylation, glycosylation, and acetylation [15]. Phosphorylation is crucial for RNA binding and replication of SARS-CoV-2. In other coronaviruses, phosphorylation of the N protein occurs predominantly during replication and transcription and decreases once the virion matures [15]. Methylation at specific arginine residues, such as R95 and R117, regulates N protein by inhibiting stress granule formation and participating in the regulation of N binding to its 5′-UTR genomic RNA [20]. N-glycosylation was observed at positions 48 and 270 of the N protein; these glycosylation sites potentially hide important epitopes and serve as a mechanism for evading the immune response [21]. Acetylation of lysine 375 has been reported previously [15].

The N protein plays many roles in the life cycle of SARS-CoV−2 (Figure 1) and mainly assembles with genomic RNA to form the viral RNA–protein complex (vRNP) [22]. These complexes facilitate binding to the viral membrane (M) protein on the surface of the ER–Golgi intermediate compartment (ERGIC) to induce budding of the vRNP complex [23]. After binding to RNA, N undergoes liquid–liquid phase separation (LLPS), forming liquid-like droplets or condensates [24, 25]. LLPS is facilitated by the linker region, which is intrinsically disordered in the N-terminal and C-terminal domain dimerization domains [26]. Phase-separated condensates participate in various biological activities, including higher-order chromatin organization, gene expression, and the triage of misfolded or unwanted proteins [27]. In the viral context, LLPS serves as a platform for optimized viral replication [28]. N undergoes LLPS and forms functional membranelle organelles to recruit TAK1 and IKK complexes, thereby promoting the activation of NF-кB [29]. Additionally, some residues in the NTD disorder region can interact with G3BP1 and modulate stress granule (SG) [30]. Furthermore, N can impair SG formation by inhibiting PKR autophosphorylation and activation and by targeting G3BP1 [31, 32]. Finally, N can localize to replication transcription complexes (RTCs) at an early stage of infection [22]. Moreover, the N protein and RNA recruit components of the RNA polymerase (RdRp) complex, such as Nsp7, Nsp8, and Nsp12, which are involved in viral gRNA replication [33, 34].

## 3. Immune Response against the Nucleocapsid Protein

**Figure 2:**
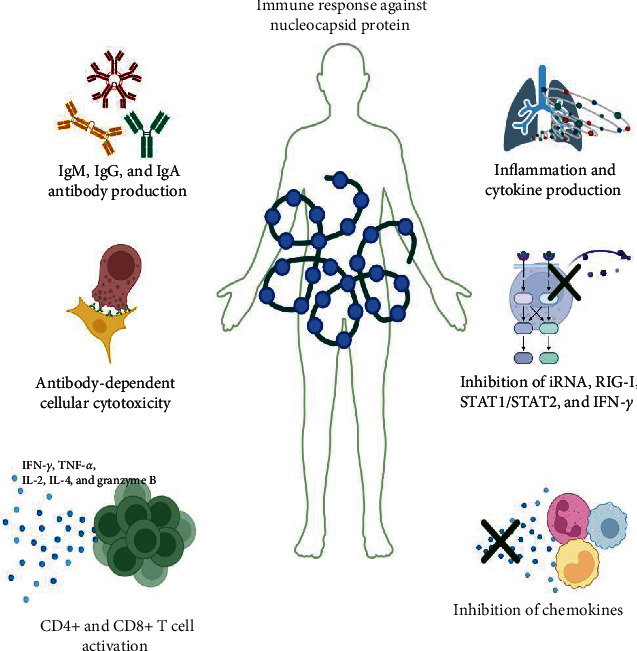
Immune response against the nucleocapsid protein. After SARS-CoV-2 infection, the nucleocapsid protein present in the body can activate or inactivate the immune system. Production of IgM, IgG, and IgA antibodies against N has been reported; also, antibody-dependent cellular cytotoxicity has been reported too. Activation of CD4+ and CD8+ T cells induces the production of different cytokines and contributes to inflammation. On the other hand, N can inhibit iRNA (interference RNA) and molecules such as RIG-I, STAT1/2, and IFN-I. Finally, N can bind to some chemokines and inhibit the recruitment of some immune cells.

The nucleocapsid proteins participate in the induction and evasion of immune responses. Intracellular N is involved in the induction of proinflammatory cytokines such as caspase-1, IL-1*β*, IL-6, and IL-18 (Figure 2) [35]. SARS-CoV-2 infection that leads to inflammasome activation and intense inflammasome formation is associated with cases of severe COVID-19 [36, 37]. N binds to gasdermin D (GSDMD) and hinders its cleavage by activated caspase-1 dimers, thus antagonizing pyroptosis [37]. Extracellular N contribute to inflammation by regulating the complement system [38, 39]. Innate immunity is triggered and regulated by the SARS-CoV-2 N protein at a very early stage of infection, and the role of the N protein in the inhibition of interfering RNA (iRNA) is relevant, since the role of iRNA involves the inhibition of viral replication [40]. Additionally, the N protein inhibits the IFN-I response by kidnapping tripartite motif-containing 25 (TRIM25), which is a ubiquitin E3 ligase involved in the antiviral innate response [41]. The N protein is also involved in the regulation of RIG-1 ubiquitination and the activation of STAT1 and 2 and consequently in their nuclear translocation [42].

Multiple tests have been developed to detect and study SARS-CoV-2 antibodies [43, 44]. Reports have demonstrated the presence of IgM, IgG, and IgA antibodies against N in the sera of SARS-CoV-2 patients, suggesting its high immunogenicity during infection and usefulness in differentiating between vaccinated and infected individuals [45]. Interestingly, several studies have shown that IgG antibodies induce antibody-dependent cell-mediated cytotoxicity (ADCC). The first studies on antibodies showed a cross-reaction between N antibodies from SARS-CoV-2 patients and N antibodies from SARS-CoV [46]. Conversely, in the context of SARS-CoV-2, antibodies elicited by the nucleocapsid protein are not only present at low concentrations but also display a notably short duration [47]. Nevertheless, memory T cells responsible for the N protein response exhibit remarkable longevity, persisting for up to 11 years post-infection [48], highlighting the suitability of the nucleocapsid protein as a valuable vaccine target and underscoring its importance in vaccine design.

### 3.1. Humoral Immune Response against the Nucleocapsid Protein

The humoral immune response elicited by N proteins remains controversial from divergent viewpoints. Some studies support the notion that N proteins induce robust antibody response, as shown in Figure 2 [23]. However, contrasting perspectives have emerged, revealing that the humoral immune response is limited and short-lived and primarily serves as an early diagnostic marker of SARS-CoV-2 infection. In another study, no differences in N antibody levels were found between COVID-19 severity groups at different ages [49]. Notably, antibody responses may not be detectable in all infected patients, particularly those with mild forms of COVID-19. In contrast, anti-N antibodies are present in SARS-CoV-2 convalescent serum [23, 24]. He et al. [45] conducted a study using PepScan analysis with overlapping peptides spanning the N protein and identified two major immunodominant epitopes located in the C-terminal (362–412 aa) and middle regions (153–178 aa). Additionally, over 75% of patients with SARS exhibit reactivity with these peptides, whereas several minor immunodominant epitopes show reactivity with approximately 50% of SARS sera [20].

Moreover, a recent study by Albecka et al. [50] indicated a correlation between N antibodies and N-specific T-cell activity within individuals, suggesting that N antibodies may offer protection against SARS-CoV-2 by promoting antigen presentation. Thus, these antibodies do not seem to possess neutralizing activity to stop viral entry into target cells. Those antibodies may participate ADCC, wherein the infected cells are coated by non-neutralizing antibodies and can be recognized through the Fc receptor by NK cells or monocytes and macrophages, neutrophils, and dendritic cells [50].

Bioinformatics analysis has revealed conserved epitopes in several human coronavirus N proteins [26]. Notably, the most immunodominant B cell epitope was located within the sequence SRGGSQASSRSSSRNSSRNSTPGSSRGTS, which spans amino acids 176–206 [26]. These findings shed light on the potential targets for future vaccine development and therapeutic interventions.

### 3.2. Cellular Immune Response against the Nucleocapsid Protein

Numerous studies have shown that SARS-CoV-2 induces a robust and long-lasting immune response, encompassing both humoral and cellular components. The strongest cellular response is triggered by N proteins [50]. Investigations into the interactions of N proteins with heparan sulfate and heparin have led to an evaluation of their potential interactions with chemokines. Lopez-Munoz et al. [19] employed biolayer interferometry (BLI) to determine N protein binding with CCL28, CCL26, CXCL9, CXCL14, CCL21, CXCL10, and CXCL-12*β*. Furthermore, in vitro assays revealed that the interaction between N and CXCL-12*β* inhibits the chemotaxis of mononuclear cells. Antibodies against N proteins activate NK cells and induce cytotoxic activity [51]. Despite its role in packing the viral RNA, the N protein should not be present on the virion surface during the viral cycle. However, recent findings have indicated that this protein is surface-exposed in infected cells after the first round of replication [19]. However, any protective immune response elicited by N proteins is likely mediated by T cells. As previously mentioned, T cells have been reported to persist for up to 11 years after infection [48]. Bert et al. [52] conducted a longitudinal study of healthy young South Asian men with mild COVID-19 humoral and cellular responses to SARS-CoV-2. They observed different kinetics in the progressive reduction of neutralizing antibodies against the N protein, whereas T-cell responses specific for various structural SARS-CoV-2 proteins remained stable over time. Cellular immune responses lasted longer than humoral responses. Interestingly, asymptomatic individuals with SARS-CoV-2 infection exhibit a more functional T-cell response than symptomatic patients [52]. Nelson et al. [53] found differences in N-specific T cells CD4+ between nonhospitalized and hospitalized individuals. In contrast, CD8+ T-cell responses to N differ according to severity but are not related to age [54, 55]. Although T cells cannot neutralize viruses, they play a crucial role in preventing severe forms of infection. Many studies have emphasized the necessity of early induction of functional SARS-CoV-2-specific T cells (IFN-*γ* secretion) in patients with mild disease to achieve rapid viral clearance [56, 57]. T-cell therapy may enhance the efficacy of current treatments against SARS-CoV-2, and cellular therapy should be designed to be as specific and directed as possible [31]. TCRs from the T cells of COVID-19 patients targeting major antigens of SARS-CoV-2, including S and N, have been identified as potential candidates for cellular therapy. Bioinformatic analysis has also been employed to map the antigenic potential of the N protein, revealing conserved motifs that can elicit cross-reactive T-cell responses [58]. Moreover, comparison of the primary B-cell and T-cell epitopes of SARS-CoV-2 with those of other human-infected coronaviruses may offer valuable insights into the development of a vaccine that provides protection against multiple coronaviruses. This information is important for potential candidates for the development of a vaccine.

## 4. Protein Spike as a Base for Vaccines against COVID-19

The humanity has fought to identify alternatives to prevent illnesses. After six centuries, vaccines have been one of the most remarkable achievements in human history, saving more lives than any other successful drug, revolutionizing life expectancy, and improving the overall quality of life. The fundamental objective of vaccination is the prevention of pathogenic infections [59].

Obstacles such as budget constraints in providing vaccines to developing countries and resistance from antivaccine groups have impeded progress in this area [60]. Furthermore, intrinsic genetic factors of pathogens, such as mutation rates and adaptability to other hosts, contribute to the complexity of eradication. One of the most relevant events in vaccinology has been the rapid and effective development of a vaccine against COVID-19, which is a disease that has recently caused 7 million of deaths. This disease is caused by SARS-CoV-2. Notably, several coronaviruses, such as SARS-CoV, MERS-CoV, and the recent SARS-CoV-2, have emerged as a result of zoonotic spillover events [8, 61]. The emergence of the COVID-19 pandemic has necessitated the rapid development of antiviral drugs and vaccines to combat infections [62]. Multiple vaccine candidates have been generated using diverse platforms, including whole virus formulations (inactivated or attenuated viruses) and nonreplicating viral vectors expressing SARS-CoV-2 antigens, nucleic acids (mRNA or DNA), and subunits (proteins or virus-like particles) [63].

Among these, the spike (S) proteins, particularly the receptor-binding domain (RBD), are prime targets for neutralizing antibodies (nAbs) [64]. Thus, S protein is the dominant vaccine target in all the COVID-19 vaccine development. It is for the fact that S protein holds a pivotal role in mediating viral entry into host cells through angiotensin-converting enzyme II (ACE-II) binding. Although most anti-COVID-19 vaccines have proven to be highly effective in inducing a protective immune response, mainly driven by nAbs targeting the S protein, the cellular immune response and potential nonsynonymous mutations in the S protein have received less attention [65]. In addition, memory B-cell responses have been observed to wane after infection, unlike memory T-cell responses, which persist for extended periods. Considering the need to enhance vaccine-induced T-cell responses, interest in alternative antigen targets is growing [48, 66]. One of the most promising candidates for inducing robust T-cell responses is the N protein [12]. Therefore, a comprehensive comparison of the main epitopes recognized by SARS-CoV-2-specific T and B cells and the immunological domains of other human-infecting coronaviruses further inform vaccine development strategies. Finally, exploring the potential of N protein-based vaccines may be key to achieving a more durable and comprehensive immune response against COVID-19 and other related coronaviruses. Thus, N protein should be considered to be included in vaccine candidate for SARS–CoV-2.

## 5. The Potential Use of Nucleocapsid as Vaccine Antigen

**Figure 3:**
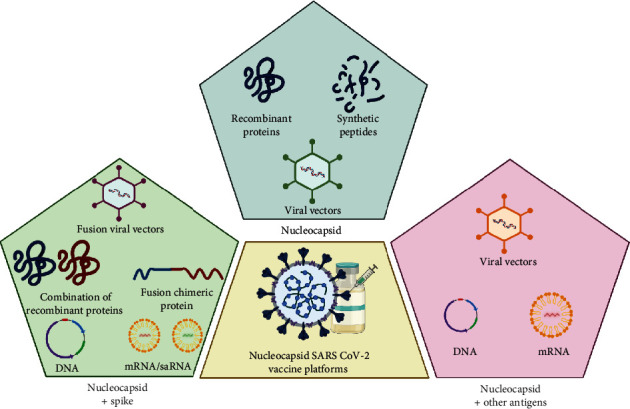
Overview of strategies in the use of nucleocapsid of SARS-CoV-2 as antigens. Immunization with nucleocapsid alone, nucleocapsid + spike, and nucleocapsid with other SARS-CoV-2 proteins has been evaluated in preclinical models.

The second generation of SARS-CoV-2 vaccines is shifting the focus toward utilizing antigens other than spike proteins. As discussed previously, the nucleocapsid protein, which elicits a robust cellular response and is highly conserved, has emerged as a promising antigen candidate [12, 48]. Numerous investigators have assessed the ability of the N protein, either alone or in combination with other antigens (Figure 3), to induce an effective and protective immune response against SARS-CoV-2 using animal models.

Interesting research work has been carried out with preclinical studies focused on evaluating the nucleocapsid protein as an antigen in the second generation of COVID-19 vaccines. In the following sections, we describe different platforms to express the N protein, different immunization routes, and doses, as well as the evaluation of different adjuvants in combination with the S protein, which of course is the main molecule that all vaccines include. Tables 1, 2, and 3 summarize these parameters and the immune response induced by the different vaccine candidates.

### 5.1. Recombinant Protein and Peptides Based on Nucleocapsid as Vaccine Candidate

Recombinant protein technology has emerged as an efficient, cost-effective, and widely available approach to facilitate production of recombinant proteins in various host expression systems, including microbial systems [60]. Several studies have used *E. coli* to express N proteins and evaluated in animal models [67, 68]. Thura et al. [69] used the complete nucleocapsid sequence of SARS-CoV-2 from Wuhan to generate a recombinant protein expressed in a prokaryotic system. Immunization of BALB/c mice with N protein resulted in the production of specific IgM, IgG1, and IgG2 antibodies. Moreover, they detected the production of various cytokines, including IL-4, IL-10, GCSF, CCL2, and TNF-*α*, and the generation of CD4+ and CD8+ memory T cells [69]. Subcutaneous immunization with other recombinant nucleocapsid protein elicited IgG antibody production in BALB/c mice [70]. The Covancell® vaccine [71], based on the nucleocapsid protein, underwent evaluation in numerous animal models and elicited both humoral and Th1/Th2 cellular responses [71]. Syrian hamsters vaccinated with Covancell® demonstrated reduced infection following the SARS-CoV-2 challenge. OVX033 [72] is a recombinant multimeric protein, N genetically fused to the self-assembling sequence OVX313 (oligoDOM®). Evaluation of this formulation in a hamster model showed T-cell responses and cross-protection against European (B.1), Delta (B.1.617.2), and Omicron (B.1.1.529) strains [72]. In addition, a microsphere formulation containing peptides from the nucleocapsid protein was evaluated in rhesus macaques [73]. Following SARS-CoV-2 challenge in both vaccinated and unvaccinated macaques, those vaccinated with N-peptides displayed a decreased viral load in the lungs. Chest radiographs of vaccinated macaques showed intact lungs, indicating potential protection. Moreover, vaccination with N peptides induced IL-2 production [73].

Presently, protein-based vaccines are being enhanced through the integration of biotechnology and nanotechnology to improve the immune responses triggered by antigens. One such development involves glyco-nanoparticles decorated with N antigens, which have shown promising immune responses in murine model [74]. As mentioned before, the principal advantage of proteins vaccines is the production; additionally, protein vaccines are more stable and can be save at 4°C [60]. However, adjuvants are required to improve induce immune responses. Although alumina is the most common adjuvant for use in human, new formulations are in development, as mentioned previously [63].

### 5.2. Viral Vectors Including Nucleocapsid Sequence and Its Potential as Vaccine

Viral vector vaccines have revolutionized the development of vaccines against infectious diseases by incorporating foreign genes that encode targeted antigens into modified viral particles [113]. The manufacturing process of the viral vector vaccines is as follows: relatively safe, easy, and scalable and induced efficient T- and B-cell responses. However, adenovirus-based viral vector vaccines can induce thrombocytopenia, and preexisting antibodies against the viral vector can reduce the immunogenicity of the vaccine [63]. Zhao et al. [114] explored the potential of SARS-CoV nucleocapsid protein to induce a protective T-cell response. Their study evaluated VSV-N353 in a mouse model as early as 2016. More recent investigations in 2021 utilized VRPs of stomatitis vesicular virus (VSV)-expressing N351 to evaluate the immune response in a mouse model [75]. Notably, mice vaccinated with VRP-351 but deprived of CD4+ or CD8+ T cells displayed similar viral loads and surveillance as unvaccinated mice following challenge with SARS-CoV-2. This underscores the significance of T-cell responses in viral clearance. Matchett et al. [76] conducted a pivotal study using an Ad5 vector expressing the entire N protein to immunize transgenic K18-h ACE-II mice and golden Syrian hamsters. Their protection against SARS-CoV-2 Wuhan and alpha strains revealed a decrease in lung viral loads in animals after challenge. Additionally, granzyme B production by CD8+ T cells has been detected, highlighting the role of the cellular immune response in clearing SARS-CoV-2 variant strain infections [76]. Another study investigated immunization with Ad5-N and subsequent boosting with the N protein, revealing the induction of antibody-dependent cellular cytotoxicity in vitro and demonstrating the functional significance of antibodies against the SARS-CoV-2 nucleocapsid [77]. Similar results have been observed with other viral vectors such as chimpanzee adenovirus (AdC) carrying a nucleocapsid [78]. Preclinical studies have consistently shown that immunization with nucleocapsid proteins induces humoral and cellular responses. These responses have potential efficacy against VOCs.  Table 1 provides a comprehensive summary of the preclinical studies evaluating immunization with nucleocapsid proteins. These findings support the significant promise of nucleocapsid-based vaccine strategies for combating SARS-CoV-2 infections and pave the way for further research and clinical development.

### 5.3. DNA Vaccines Coding for the Nucleocapsid Protein

DNA vaccines have gained significant attention and success against viruses such as HIV, Ebola, and HPV, mainly because of their relatively cheap, rapid, and scalable production [46]. Compared with mRNA vaccines, DNA vaccines have higher stability and can be stored for a long time. However, in most cases, intramuscular or electroporation injections are required [63]. In 2023, Manfredi et al. [79] designed a DNA coding for nucleocapsid protein fused to C-terminus of HIV Nef protein and evaluated the immune response in K18-h ACE-II mice. Vaccinated mice show mainly a CD8+ T cell response and production of cytokines such as IFN-*γ*, IL-2, and TNF-*α*. Furthermore, these animals survived after the challenge with SARS-CoV-2 and showed a decrease in viral loads in lungs. Even though the addition of the C-terminus of HIV to the sequence of nucleocapsid allows the incorporation into extracellular vesicles, the authors propose that vaccination with this antigen induces a systemic and protective immune response [79]. Preclinical studies evaluating the nucleocapsid protein during immunization are summarized in Table 1.

### 5.4. Vaccine Candidate that Includes Nucleocapsid and Spike Proteins

**Figure 4:**
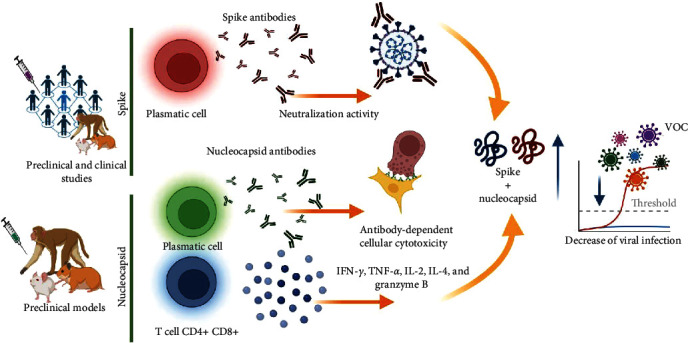
Immune response induced by the combination of SARS-CoV-2 spike and nucleocapsid proteins. Overview of immune response induce by spike and nucleocapsid protein. Preclinical studies indicated that the combination of spike and nucleocapsid increases humoral and cellular immune response and is effective against VOCs.

Numerous reports on SARS-CoV-2 and MERS-CoV have consistently demonstrated that antibodies generated against the spike proteins exhibit potent neutralizing activities [8, 115, 116]. Consequently, the spike protein of SARS-CoV-2 has been incorporated into over 90% of the vaccines being developed [117]. Researchers have explored the combination of S and N proteins or sequences encoding for the mentioned proteins, as a promising strategy for enhancing humoral and cellular immune responses (Figure 4). This approach has considerable potential for eliciting robust and comprehensive immune responses, making it a key focus in ongoing efforts to combat these viral infections.

### 5.5. Combination of Nucleocapsid and Spike Recombinant Proteins Induces Immune Response

An intriguing approach, combining the N protein with subunit S1 or S2 of the spike protein utilizing BALB/c mice demonstrated a higher induction of specific S1, S2, and N IgG antibodies and neutralizing activity and IFN-*γ* production in all immunized animals [80]. Hong et al. [81] investigated the combination of the N protein with the S protein using an *E. coli* expression system, wherein they fused the RBD of the spike protein with the tetanus toxoid epitope P2 and the whole N protein. Immunization induced IgG1 and IgG2 antibodies against N and RBD, along with IFN-*γ* production. Interestingly, higher antibody and IFN-*γ* levels were observed with the combination involving the N protein. Furthermore, the combination of subcutaneous RBD + N was evaluated in a BALB/c mouse model, resulting in antibody production and the production of cytokines such as IFN-*γ*, granzyme B, IL-4, and IL-12 [82]. Formulation of nucleocapsid and prefusion-full S protein (SFL_mut_) in combination with flagellin (KF) and cyclic GMP-AMP (cGAMP) elicited stronger systemic and mucosal humoral immunity in a mouse model [83]. Although prokaryotic expression systems are widely used, they lack posttranslational modifications, such as glycosylation [118]. To address this, combinations of S1 and RBD with nucleocapsid proteins expressed in eukaryotic systems have been investigated. Immunization with S1 + N or RBD + N induced IgG1, IgG2, and neutralizing activity, and production of IFN-*γ*, TNF-*α*, and IL-2 with higher responses was observed in combination with the N protein. Moreover, sera from the immunized mice showed cross-reactivity with RBD from Alpha and Beta VOCs [84]. A combination of spike and nucleocapsid proteins using a conventional formulation with aluminum hydroxide, lipids, or other adjuvants has shown induction of humoral and cellular response [85, 86, 87, 88, 89]. Moreover, formulation of spike + nucleocapsid with cationic or anionic lipids showed that this combination of antigens induced IgG and IgA antibodies and production of IL-5 and IFN-*γ* [86]. Biotechnological approaches have also been employed, such as the generation of exosomes decorated with spikes (STX-S) or nucleocapsids (STX-N) [90]. These approaches led to improved immune responses, particularly with the combination of S + N. Another strategy in development involves fusing the most immunogenic regions of different SARS-CoV-2 proteins, resulting in a protein fusion called “SpiN” [91]. Evaluation of SpiN in K18-h ACE-II mice showed that antibodies induced by vaccination lacked neutralization activity, but the immunized animals survived challenges with the SARS-CoV-2 Wuhan, Delta, and Omicron variants. This survival was not observed when vaccinated mice were deprived of CD4+ and CD8+ T cells, emphasizing the importance of the cellular response in clearing SARS-CoV-2 infection [91]. Mambelli et al. [92] developed a chimeric protein fused to rBCG, incorporating the immunogenic regions of S and N proteins. Their study in K18-h ACE-II mice revealed the termination of both humoral and cellular immune responses and induction of neutralizing antibodies against Gamma VOC [92]. Additionally, Lam et al. [93] propose a spike/nucleocapsid such as a booster for approved vaccines. In this study, Balb/c mice were immunized with two doses of the BioNTech COVID-19 vaccine and received an intranasal booster with N-RBD fusion protein. Immunized mice showed neutralization activity against D614G, Delta, and Omicron SARS-CoV-2 VOCs [93]. These studies provide evidence for the utility of N protein in the induction of an immune response against certain SARS-CoV-2 VOCs. Moreover, the use of specific regions of S and N proteins has been proven effective in eliciting an immune response. These findings highlight the potential of innovative approaches using N proteins to enhance vaccine strategies against SARS-CoV-2 (Figure 4).

### 5.6. Immunization with Combination of Viral Vectors Containing N and S Encoding Sequences

Dangi et al. [94] evaluated individual and combination vectors expressing the S or N proteins. They immunized K18-h ACE-II mice with an adenoviral vector expressing either the SARS-CoV-2 spike (Ad5-S), nucleocapsid (Ad5-N), or both (Ad5-S + Ad5-N) and subsequently challenged the mice with SARS-CoV-2. Notably, the groups immunized with Ad5-S and Ad5-S + Ad5-N showed decreased viral genome levels. Only the group immunized with Ad5-S + Ad5-N showed a decrease in the viral genome in the brain, suggesting that the combination of S and N may contribute to the clearance of distal SARS-CoV-2 infection [94]. Rice et al. [95]. developed a vector viral vaccine using hAd5 S-fusion + N-ETSD, expressing the SARS-CoV-2 spike-fusion form and nucleocapsid fused to ETSD. Immunization with hAd5/S-fusion-N-ETSD in CD-1 mice resulted in the induction of IgG1, IgG2a, IgG2b, and IgA antibodies and the production of cytokines such as IFN-*γ*, TNF-*α*, and IL-2. Remarkably, higher cytokine responses were observed in groups receiving a combination of subcutaneous and intranasal immunizations, indicating the utility of heterologous immunization in enhancing the immune response [95]. This group also evaluated Ad5 S-fusion + N-ETSD in rhesus macaques, observing a decrease in viral load in both lung and nasal swabs in all immunized macaques [96]. Modified vaccinia Ankara (MVA) hase been used to develop COVID-19 vaccines [97, 98, 99, 100, 101]. Chiuppesi et al. [98] used a modified vaccinia Ankara (MVA) vector platform to produce synthetic MVA (sMVA) vectors coexpressing SARS-CoV-2S and N antigens. Continuing their investigation, they evaluated sMVA-N/S in hamsters and nonhuman primates (NHPs) [99]. Sera from immunized hamsters demonstrated neutralizing activity against VOCs D614, Alpha, Beta, Gamma, and Delta. Additionally, sera from immunized NHPs were capable of recognizing spike proteins from the Beta, Gamma, and Delta strains, as assessed by ELISA. Zhong et al. [100] developed an MVA-based vaccine. Mice immunized with MVA-S + N exhibited induced spikes and nucleocapsid IgG and IgA antibodies. Although no neutralizing activity was detected, mice challenged with mouse-adapted SARS-CoV-2 [119] displayed viral clearance, attributable to the cellular response induced by intranasal vaccination with MVA-S+N [100]. Using another MVA-expressing S and N protein based on the Wuhan, Beta, or Omicron BA.1 strains, the clearance of infections by Omicron subvariants was determined using a hamster model [101]. Recently, Clever et al. [102] developed another recombinant MVA vaccine coexpressing SARS-CoV-2 prefusion-stabilized spike protein (ST) and SARS-CoV-2 nucleoprotein. Protection in K18-h ACE-II mice was observed. A novel vaccinia virus (VACV) coexpressing the spike and nucleocapsid proteins (rACAM2000SN) induced antibodies with neutralization activity and reduction of SARS-CoV-2 viral loads in a hamster model [103]. Additionally, immunization with VSV expressing S and N resulted in decreased infection against Alpha, Beta, and Delta VOCs [104]. Collectively, these studies provide evidence for the utility of N in dealing with SARS-CoV-2 VOCs.

### 5.7. RNA and DNA Immunization with the Combination of S and N Encoding Sequences of RNA Vaccine Candidates

As we mentioned before, DNA vaccines for many viruses have been developed and evaluated in preclinical models. Consistent with this, Ahn et al. [105] developed a plasmid encoding the spike protein (GX-19) and another plasmid encoding the RBD and N proteins (GX-19N) of SARs-CoV-2. The vaccines were evaluated in mice and NHPs; DNA immunization induced antibodies with neutralization activity and IFN-*γ* production, with GX-19N showing a particularly robust response. The efficacy of immunization with this plasmid has been previously demonstrated and is currently being evaluated in clinical trials (NCT04715997).

The development of RNA-based vaccines has marked a new era in the fight against SARS-CoV-2, with Moderna and Pfizer leading the development and administration of vaccines worldwide. The efficacy, safety, and security of these vaccines have been rigorously determined [120, 121]. In addition, RNA COVID-19 vaccines induce strong Th1- and B-cell responses and produce long-lived plasma and memory B cells. However, because of the instability of RNA, RNA vaccines need to be stored at a lower temperature, which puts forward certain requirements for the storage environment of the inoculation unit [63]. McCafferty et al. [106] developed a self-amplifying RNA (saRNA) vaccine encoding SARS-CoV-2 spike-RBD and N antigens. When administered in a formulation called ZIP1642, sera from immunized mice showed neutralizing activity against various VOCs, including Wuhan, Beta B.1.351, Delta B.1.617.2, and Omicron B.1.1.529. Immunized Syrian hamsters exhibited decreased lung viral loads and no organ damage after SARS-CoV-2 challenge [106]. Similarly, Hajnik et al. [107] designed mRNAs expressing the SARS-CoV-2 full-length nucleocapsid (mRNA-N) or prefusion-stabilized S protein with two proline mutations (mRNA-S-2P). Syrian hamsters immunized with this combination displayed robust humoral and cellular immune responses and were protected against challenge with Delta and Omicron VOCs [107]. Preclinical data from studies involving the nucleic acids encoding the N protein strongly support the use of this antigen as a promising strategy to combat SARS-CoV-2 VOCs. Preclinical studies evaluating the combination of S and N proteins during immunization are summarized in Table 2.

## 6. Nucleocapsid and Other Antigens as Target for SARS-COV-2 Vaccines

In addition to the use of N in combination with the S protein, researchers have explored the combination of N with other structural, nonstructural, and accessory proteins of SARS-CoV-2 in preclinical models (Figure 3). Current DNA vaccines incorporate membrane proteins in combination with N and other SARS-CoV-2 proteins. For instance, combinations of DNA plasmids coding for M, E, and N have been evaluated in cynomolgus macaques [108], whereas DNA coding for N, M, and RBD from various VOCs has been assessed in BALB/c and K18-h ACE-II mice [109], thus providing potential strategies to address variants such as Beta, Delta, and Omicron. Nonstructural proteins of SARS-CoV-2, such as NSP12, have also been evaluated in combination with S and N [110]. In the mRNA vaccine realm, the BNT162b4 vaccine (N + M + NSPs) was evaluated in combination with the mRNA vaccine BTN162b2 to enhance the immune response against VOCs [111], and the combination of BNT162b4 with BTN162b2 is currently in the clinical phase (NCT 05541861) [111]. Additionally, immunization with lentiviruses carrying peptides of S, N, and ORF1 has shown promise in K18-h ACE-II mice, demonstrating protection and cross-reactivity with Omicron VOC [112]. Preclinical studies evaluating combinatorial immunization of N with other SARS-CoV−2 proteins are summarized in Table 3.

## 7. Concluding Remarks

Since the outbreak of SARS-CoV in 2003, extensive research has been conducted on the biology and immune responses associated with the nucleocapsid protein. These investigations revealed remarkable conservation of this protein across various coronaviruses, demonstrating its potential as a target for vaccine development. Specifically, the nucleocapsid protein of SARS-CoV-2 has shown high sequence conservation, even among the different VOCs, making it an ideal candidate for inclusion in second-generation COVID-19 vaccines. Immunization with the SARS-CoV-2 nucleocapsid induces both humoral and cellular immune responses, with evidence suggesting long-lasting immunity. However, it is important to note that current COVID-19 vaccines do not confer sterilizing immunity or long-term protection, highlighting the need to incorporate additional antigens into vaccine formulations. Notably, the combination of nucleocapsids and spike proteins has emerged as a promising strategy to elicit both neutralizing antibodies and robust cellular immune responses. Numerous studies have demonstrated that this combination leads to a heightened immune response, resulting in protection against various VOCs.

Although the exact duration of the immune response induced by nucleocapsid immunization remains to be fully elucidated, based on the findings from SARS-CoV research, it is plausible that the nucleocapsid-induced cellular immune response may persist over the long-term. In conclusion, the nucleocapsid protein represents an excellent antigen for inducing immune responses, and its combination with the spike protein has proven to be a potential strategy for enhancing the immune response and effectively tackling variants of SARS-CoV-2. Continued efforts in this direction hold great promise in improving the efficacy and durability of COVID-19 vaccines.

## Figures and Tables

**Table 1:** Overview of published preclinical studies that used the nucleocapsid protein of SARS-CoV-2 as antigen.

	Platform	Animal model	Antigen	Dose and route	Immune response	Protection	Cross-reaction with VOC	Ref
1	Protein, P.S.	Wistar rats	Whole nucleocapsid	Fourth 150 *µ*g IM	IgG Ab	UD	UD	[67]
2	Protein, P.S.	BALB/c mice	Whole nucleocapsid	Two 50 *µ*g IM	IgM and IgG Ab IFN-*γ*	UD	UD	[68]
3	Protein, P.S.	BALB/c mice	Whole nucleocapsid	Fourth 75 *µ*g, IP	IgM and IgG Ab IFN-*γ*, CCL2, GCSF, IL10, CCL5, TNF IR, and TNF-*α*	UD	UD	[69]
4	Protein, P.S.	BALB/c mice	Whole nucleocapsid	Three, 10 *µ*g SC	IgG Ab	UD	UD	[70]
5	Protein, P.S.	BALB/c mice Syrian hamster Rabbits *Callithrix* *jacchus* monkeys	Whole nucleocapsid	Two 50 *µ*g IM	IgG Ab IFN-*γ*, TNF-*α*, IL-4, and IL-5	Positive	UD	[71]
6	Protein, P.S.	Syrian hamster	Whole nucleocapsid fused to OVX313 protein	Two 50 *µ*g IM	IgG Ab IFN-*γ*	Positive	Europe, Delta, and Omicron	[72]
7	Synthetic peptides	Rhesus macaques	CTL epitopes of nucleocapsid	Thrice 20 mg LN and 100 mg IT	IL-2, IL17 B, and CX3CL1	Positive	UD	[73]
8	Protein	C57BL/6 mice	Glyconanoparticles decorated with nucleocapsid	Two, 50 *µ*g, SC	IgG Ab CTL activity	UD	UD	[74]
9	Viral vector VSV	BALB/c and C57 mice	353 N peptide	Two, 10^5^ VRP-N353, and IN	IFN-*γ*, TNF*α*, IL-10, CD44, CD43, CD11a, CD27, CXCR6, CTLA4, and PD-1	Positive	UD	[75]
10	Viral vector Ad5	Syrian hamster	Whole nucleocapsid	One, 1.8 ×10^11^ VP Ad5-N. IV	Granzyme B	Positive	Alpha	[76]
11	Viral vector Ad5 and protein	K18-h ACE-II mice	Whole nucleocapsid	Two first 10^11^ PFU Ad5, second 50 *µ*g of N, IM	IgG Ab CD107a	Positive	UD	[77]
12	Viral vector AdC6 and ADC7	C57BL/6 mice	Nucleocapsid fused to gD-HSV1	Two, 5 × 10^10^ VP, IM	IFN-*γ* and CD44+	UD	UD	[78]
13	DNA	K18-h ACE-II mice	Nucleocapsid fused to Nef protein HIV	Two 10 *µ*g IM	IFN-*γ*, IL-2, and TNF-*α*	Positive	UD	[79]

IP, intraperitoneal; IM, intramuscular; IT, intratracheal; SC, subcutaneous; IV, intravenous; LN, lymph node; UD, undetermined; PS, prokaryotic system.

**Table 2:** Overview of published preclinical studies that combined the nucleocapsid and spike protein of SARS-CoV-2.

	Platform	Animal model	Antigen	Dose and route	Immune response	Protection	Cross-reaction with VOC	Ref
1	Protein, P.S.	BALB/c	S1 + N S2 + N	Two, 25 *µ*g S1/S2 + 25 *µ*g N, IP	IgG and IgG2 Ab IFN-*γ*	UD	UD	[80]
2	Protein, P.S.	BALB/c and C57BL/6 and K18-h ACE-II mice Rats Cambodian macaques	RBD fused tetanus toxoid epitope P2 (RBD-P2) + nucleocapsid	Two, IM 30 *µ*g and 50 *µ*g RBD-P2 3 *µ*g and 3 *µ*g of N	IgG1 and IgG2 Ab IFN-*γ* and IL-4 CD69+, CD62L, and CD44+	Positive	UD	[81]
3	Protein, P.S.	BALB/c mice	RBD + N	15 *µ*g RBD + 15 *µ*g N. SB	IgG1 and IgG2a Ab IFN- *γ*, granzyme B, IL-4, and IL-12	UD	UD	[82]
	Protein, P.S.	C57BL/6 and K18-h ACE-II mice	N + SFL_mut_	8 *µ*g NC + 8 *µ*g SFL_mut_ IN	IgG and IgA Ab IFN-*γ* and IL-4 Tissue-resident memory cells	Positive	B.1351 and B.1.617.2	[83]
4	Protein, E.S.	BALB/c mice	S1 + N RBD + N	Three, 10 *µ*g S1/RBD + 10 *µ*g N, IP	IgM, IgG1, and IgG2a Ab IFN-*γ*, TNF-*α*, and IL-2	UD	Alpha and Beta	[84]
5	Protein, E.S	BALB/c mice	N + RBD	Three, 50 *µ*g SC or IN	IgG1, IgG2a, and IgA Ab	UD	Delta	[85]
6	Protein, E.S	C57BL/6 mice	S1 + N	Two, S1 + N 0.1/1 *µ*g IN	IgG, IgA, and Ab IFN-*γ* and IL-5	UD	UD	[86]
7	Protein, E.S.	K18-h ACE-II mice and BALB/c mice	S1 + S2 + N	Two S1 + S2 10 *µ*g N 2.5 *µ*g IP	IgG Ab IFN-*γ*	Positive	UD	[87]
8	Protein E.S. and E.S	BALB/c mice	RBD + N	Two, 10 *µ*g RBD + 10 *µ*g N IM	IgG Ab IFN-*γ*	UD	D614G and Delta	[88]
9	Protein, E.S.	BALB/c mice	S1 + N RBD + N	Three, S1 + N or RBD + N 40, 80, or 120 *µ*g	IgG Ab IFN-*γ*, IL-4, and IL-12	UD	UD	[89]
10	Protein, E.S.	BALB/c mice Rabbits	Exosome decorated with S + exosome decorated with N	One 25 ng S + 5 ng NC, 125 ng S + 10 ng N IM	IgG Ab IFN-*γ*	UD	Delta and Omicron	[90]
11	Protein, P.S.	K18-h ACE-II mice Syrian hamster	Fusion protein S and N (SpiN)	Two, 10 *µ*g SpiN, IM	IgG Ab IFN-*γ* CD44+, CD69+, and CD62L+	Positive	Omicron	[91]
12	Protein, P.S.	K18-h ACE-II mice	Chimeric protein Spike and N fused to BCG (rBCG-ChD6)	Two, 10^6^ CFU rBCG-chD6. SC	IgG1 and IgG2c Ab IFN-*γ* and IL-6	Positive	Gamma	[92]
13	Protein, E.S.	BALB/c mice	Fusion protein nucleocapsid RBD	Booster N-RBD, 18 *µ*g. IN	IgG Ab	UD	D614G, Delta, and Omicron	[93]
14	Viral vector Ad5	K18-h ACE-II mice	Spike + nucleocapsid	One, 10^9^ PFU Ad5-N + S	IgG Ab IFN-*γ*	Positive	UD	[94]
15	Viral vector Ad5	CD-1 mice	Spike-fusion + nucleocapsid fused to ETSD	Two, 10^9^ VP. SC and IN	IgG1. IgG2a, and IgG2b IFN-*γ*	UD	UD	[95]
16	Viral vector Ad5	Rhesus macaques	Spike-fusion + nucleocapsid fused to ETSD	Three, 10^11^ VP hAd5 S-fusion + N-ETSD. SC and O	IgG Ab IFN-*γ*	Positive	UD	[96]
17	Viral vector MVA	Rhesus macaques	Spike stabilize (SdFCS) + nucleocapsid	Two, 10^8^ PFU MVA-N/SdFCS-N, BU, IM, and SL	IgG Ab IFN-*γ*	Positive	Delta	[97]
18	Viral vector MVA	BALB/c and C57BL/6 Mice	Spike + nucleocapsid	Two, 10^7^ PFU. MVA-N/S, and IP	IgG Ab IFN-*γ*, TNF-*α*, and IL-4	UD	UD	[98]
19	Viral vector MVA	Syrian hamster and rhesus macaques	Spike + nucleocapsid	Two, 1 × 10^8^ PFU or 2.5 × 10^8^ MVA-N/S, IM or IN	IgG Ab IFN-*γ*, IL-2, IL-4	Positive	Alpha, Beta, Gamma, and Delta	[99]
20	Viral vector MVA	BALB/c mice	Spike + nucleocapsid	Two, 1 × 10^7^ PFU or 2.5 × 10^8^ MVA-N/S, IM, or IN	IgG and IgA Ab IFN-*γ* and granzyme B CCL2, CXCL10, CCL3, TNF-*α*, and IL-6	Positive	UD	[100]
21	Viral vector MVA	Syrian hamster	Spike + nucleocapsid	Two, 1 × 10^8^ PFU or 2.5 × 10^8^ sMVA-N/S, IM	IgG Ab	Positive	Beta and Omicron	[101]
22	Viral vector MVA	K18-h ACE-II mice	Spike + nucleocapsid	One, 1 × 10^8^ PFU MVA-ST/N, IM	IgG Ab IFN-*γ*	Positive	UD	[102]
23	Viral vector VACV	Syrian hamster	Spike + nucleocapsid	One, 2 × 10^7^ PFU VACV N + S (rACAM2000SN) IM	IgG Ab	Positive	UD	[103]
24	Viral vector VSV	Syrian hamster	Spike + nucleocapsid	One, 1 × 10^5^ PFU VSV-SARS2-N+S IM or IN	IgG Ab	Positive	Alpha, Beta, and Delta	[104]
25	DNA	BALB/c mice Cynomolgus monkey	S-RBD + nucleocapsid (GX-19 N)	Two, 12 *µ*g GX-19 N, IM Two, 3 mg GX-19 N, IM	IgG Ab IFN-*γ*	UD	UD	[105]
26	RNA SaRNA	Swiss mice Syrian hamster	RBD + nucleocapsid	Two, 0.5 *µ*g S-RBD saRNA + 0.5 *µ*g N saRNA (ZIP1642) IM	IgG Ab IFN-*γ*, TNF-*α*, IL-2, and IL-4	Positive	Beta and Delta	[106]
27	RNA MRNA	BALB/c mice Syrian hamster	Spike + nucleocapsid	Two, 1 *µ*g mRNA-S + 1 *µ*g mRNA-N IM Two, 2 *µ*g mRNA-*S* + 2 *µ*g mRNA-N IM	IgG Ab IFN-*γ*, TNF-*α*, and IL-2	Positive	Delta and Omicron	[107]

IP, intraperitoneal; IM, intramuscular; IT, intratracheal; SC, subcutaneous; IV, intravenous; SL, sublingual; BU, buccal; LN, lymph node; UD, undetermined; procaryotic system; ES, eukaryotic system.

**Table 3:** Overview of published preclinical studies that combined nucleocapsid with other antigens of SARS-CoV-2.

	Platform	Animal model	Antigen	Dose and route	Immune response	Protection	Cross-reaction with VOC	Ref
1	DNA/viral vector pcDNA and SeV	Cynomolgus macaques	Nucleocapsid + membrane + envolve pcDNA/NME	Two, pcDNA/NME boost with SeV-NME. IM	IFN-*γ*	Positive	UD	[108]
2	DNA	BALB/c and h ACE-II mice	RBD VOCS, nucleocapsid and membrane	Three, 50 *µ*g, IM	IgG Ab IFN-*γ*	Positive	Beta, Delta, and Omicron	[109]
3	Viral vectors HuAd and ChAd	BALB/c and h ACE-II mice	S1 domain, nucleocapsid and RdRp	One, 5 × 10^7^ PFU, IM or IN	IgG and IgA Ab IFN-*γ*	Positive	Alpha and Beta	[110]
4	RNA mRNA	HLA-A2.1 transgenic mice Syrian hamsters	Nucleocapsid, membrane and NSPs	Two, 3 *µ*g BNT162b4	IFN-*γ* CD107a	Positive	Delta and Omicron BA.1	[111]
5	Viral vector lentiviral	h ACE-II-KI mice	Peptides of spike, nucleocapsid and ORF1	Two, 1 × 10^6^ transduced cells. IV	IFN-*γ* and TNF-*α*	Positive	Omicron	[112]

IP, intraperitoneal; IM, intramuscular; IT, intratracheal; SC, subcutaneous; IV, intravenous; LN, lymph node; UD, undetermined.

## Additional Points

*Limitation of the Study*. Data on the immune responses induced by different N vaccines are limited to preclinical studies. Some of these candidates are now in the clinical phase; however, the results have not been discussed. Results in humans are not yet available; therefore, this discussion is based on data obtained from animal models.
